# Identification of CDK2 substrates in human cell lysates

**DOI:** 10.1186/gb-2008-9-10-r149

**Published:** 2008-10-13

**Authors:** Yong Chi, Markus Welcker, Asli A Hizli, Jeffrey J Posakony, Ruedi Aebersold, Bruce E Clurman

**Affiliations:** 1 Divisions of Clinical Research and Human Biology, Fred Hutchinson Cancer Research Center, 1100 Fairview Avenue N., Seattle, WA 98109, USA; 2 Institute for Systems Biology, 1441 N. 34th Street, Seattle, WA 98103, USA; 3 Division of Basic Sciences, Fred Hutchinson Cancer Research Center, 1100 Fairview Avenue N., Seattle, WA 98109, USA; 4 Institute of Molecular Systems Biology, ETH Zurich and Faculty of Science, University of Zurich, 8093 Zurich, Switzerland

## Abstract

**Background:**

Protein phosphorylation regulates a multitude of biological processes. However, the large number of protein kinases and their substrates generates an enormously complex phosphoproteome. The cyclin-dependent kinases - the CDKs - comprise a class of enzymes that regulate cell cycle progression and play important roles in tumorigenesis. However, despite intense study, only a limited number of mammalian CDK substrates are known. A comprehensive understanding of CDK function requires the identification of their substrate network.

**Results:**

We describe a simple and efficient approach to identify potential cyclin A-CDK2 targets in complex cell lysates. Using a kinase engineering strategy combined with chemical enrichment and mass spectrometry, we identified 180 potential cyclin A-CDK2 substrates and more than 200 phosphorylation sites. About 10% of these candidates function within pathways related to cell division, and the vast majority are involved in other fundamental cellular processes. We have validated several candidates as direct cyclin A-CDK2 substrates that are phosphorylated on the same sites that we identified by mass spectrometry, and we also found that one novel substrate, the ribosomal protein RL12, exhibits site-specific CDK2-dependent phosphorylation *in vivo*.

**Conclusions:**

We used methods entailing engineered kinases and thiophosphate enrichment to identify a large number of candidate CDK2 substrates in cell lysates. These results are consistent with other recent proteomic studies, and suggest that CDKs regulate cell division via large networks of cellular substrates. These methods are general and can be easily adapted to identify direct substrates of many other protein kinases.

## Abstract 2

Engineered kinases and thiophosphate enrichment were used to identify many candidate CDK2 substrates in human cell lysates.

## Background

Reversible protein phosphorylation is one of the most common posttranslational modifications and regulates virtually all cellular processes. Protein kinases are among the largest known gene families with more than 500 human kinase genes that comprise nearly 2% of the open reading frames of the human genome [1]. Moreover, approximately 30% of all cellular proteins are phosphorylated [2]. The large number of kinases and their substrates make it very difficult to determine which proteins are phosphorylated by specific kinases *in vivo*, but this information is critical to understanding kinase functions and the control of biological processes in general. Various strategies have been developed to identify protein kinase substrates, and several have resulted from recent technological advances in substrate detection.

Some approaches have utilized antibodies against phosphomotifs within the substrate proteins as affinity reagents to enrich for phosphorylated peptides. Examples include using antibodies that recognize conserved motifs that are highly specific to a particular kinase [3], as well as the use of phosphomotif antibodies combined with changes in physiological conditions that stimulate kinase function [4]. However, it is difficult to apply these approaches to kinases that phosphorylate broader substrate motifs, since there is less epitope conservation among substrates. Another recent method combined quantitative phosphoproteomics with kinase knock-outs and cellular perturbations to identify kinase targets in yeast [5]. However, with these cell-based approaches, it is often difficult to determine if the putative substrates are direct kinase targets. An *in vitro *approach employing arrays of proteins phosphorylated by isolated recombinant kinases has been successfully used in a global analysis of yeast kinase substrates, but this strategy may be difficult to apply to organisms with larger proteomes [6].

A 'chemical genetic' approach developed by Kevan Shokat's laboratory addresses many of these potential problems [7]. In this technique, the kinase to be studied is mutated by replacing a conserved bulky residue within the ATP-binding pocket with a smaller residue. This creates an enlarged ATP binding pocket that enables the mutant kinase to utilize bulky ATP analogues that cannot be used by wild-type cellular kinases, thereby isolating the activity of the mutant kinase from all other cellular kinases [8]. This technique is broadly applicable to most protein kinases and has led to important advances in substrate identification, including the description of a large number of potential cyclin-dependent kinase (CDK) substrates in yeast [9-15]. However, several technical hurdles add substantial challenges when applying this approach to mammalian kinases with broad substrate networks.

In this study, we report a method employing the Shokat strategy to identify direct cyclin A-CDK2 substrates in human cell lysates. CDK2 is activated by both the cyclin E and cyclin A subunits, and cyclin A-CDK2 plays critical roles in cell cycle control, primarily in G1 and S-phases [16,17]. We used an engineered cyclin A-CDK2 and ATP-γ-S analogue to label proteins with thiophosphates in cell lysates, and after digestion of the protein mixtures, we employed a single-step chemical enrichment procedure to selectively isolate thiophosphorylated peptides. As these studies were nearing completion, Blethrow *et al*. [18] independently reported a similar approach employing engineered CDK1 and thiophosphate enrichment methods that they used to identify a group of 68 putative cyclin B-CDK1 substrates within Hela cell lysates.

We identified 180 proteins and over 220 phosphopeptides that were phosphorylated in cell lysates by cyclin A-CDK2, and these proteins represented diverse cellular pathways. To validate these methods, we selected several candidate substrates and confirmed that they were phosphorylated by cyclin A-CDK2 *in vitro *on the same sites that we identified in the screen. Finally, we selected one novel substrate, the ribosomal protein RL12, for further study: site-directed mutagenesis and phosphopeptide mapping confirmed that CDK2 phosphorylates RL12 *in vitro *and *in vivo *on the same site determined by our methods.

## Results and discussion

### Utilization of ATP analogues by engineered CDKs

**Figure 1:**
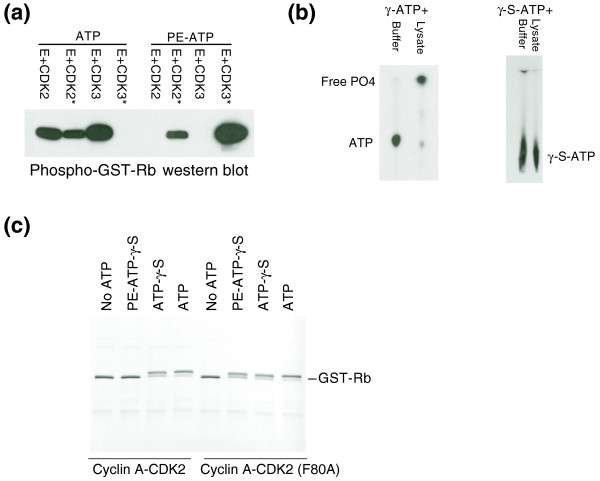
Characterization of the engineered CDKs. **(a) **Cyclin E-CDK2/3 complexes, or their F80A engineered counterparts (indicated by asterisks), were immunoprecipitated from transfected U2OS cell lysates via the HA-tag on the CDK subunit and subjected to *in vitro *kinases assays with 10 μM of either normal ATP or PE-ATP analogue. Phosphorylation of GST-Rb was monitored by immunoblotting with a phosphospecific anti-pS780-Rb antibody (New England Biolabs). The wild-type kinases cannot use PE-ATP. **(b) **Thin-layer chromatography - analysis reveals hydrolysis of ATP in cell lysate and transfer to acceptor nucleotides (left). The positions of the free phosphate and ATP are indicated. In contrast, ATP-γ-S is not hydrolyzed in cell lysates (right). **(c) **Kinase assays were performed using wild-type and cyclin A-CDK2 (F80A) complexes purified from *E. coli *and GST-Rb in the presence of 200 μM of ATP, ATP-γ-S and PE-ATP-γ-S at room temperature for 2 h. Kinase reactions were analyzed by SDS gel electrophoresis and visualized by Coomassie staining. Extent of the GST-Rb phosphorylation was monitored by the electromobility shift of GST-Rb.

We generated mutant 'Shokat' CDKs containing amino acid exchanges at a conserved bulky residue in their ATP binding pockets. In the case of CDK2 and CDK3 this was a phenylalanine to alanine exchange at position 80, designated CDK2 (F80A). We also synthesized 12 ATP analogues to determine if the engineered CDKs can use these analogues to phosphorylate recombinant Retinoblastoma (Rb) protein *in vitro*, and found that they utilized *N*^6^-(2-phenylethyl)-ATP (PE-ATP) most efficiently (Figure 1a, and data not shown). Although both wild-type and cyclin E-CDK2 (F80A) used normal ATP to phosphorylate glutathione-S-transferase (GST)-Rb protein, only the F80A kinase could use PE-ATP. Similar results were obtained with cyclin E-CDK3, but in this case the F80A mutant could no longer use normal ATP, although the structural basis for this observation is unclear (Figure 1a). These studies confirmed that the wild-type and engineered kinases exhibit the desired ATP specificities.

Whereas the mutant CDKs efficiently used PE-ATP to phosphorylate Rb *in vitro*, similar experiments utilizing radiolabeled PE-ATP in cell lysates failed because the labeled phosphate was cleaved from PE-ATP by an ATPase activity in the lysates (data not shown). We therefore switched to the thiophosphate form of the ATP analogue (PE-ATP-γ-S), which was not hydrolyzed by lysates (Figure 1b). Although kinases often use ATP-γ-S less efficiently than normal ATP, thiophosphorylation has several advantages in this context. First, thiophosphates are more stable and resistant to phosphatases [19]. Second, since there are no pre-existing thiophosphorylation events in the cells, thiophosphate labeling provides unique markers for proteins phosphorylated by the mutant kinase. Finally, the thiophosphate group has similar chemical properties as the sulfhydryl group and is amenable to chemical modifications. We expressed and purified soluble wild-type and cyclin A-CDK2 (F80A) complexes from bacteria and carried out a similar Rb kinase assay to test their ability to use PE-ATP-γ-S. As shown in Figure 1c, although both kinases can use ATP or ATP-γ-S to phosphorylate GST-Rb protein (as indicated by its electromobility shift) only the F80A mutant can use PE-ATP-γ-S. We thus used PE-ATP-γ-S for all of our subsequent studies.

### Single-step purification of thiophosphorylated peptides

**Figure 2:**
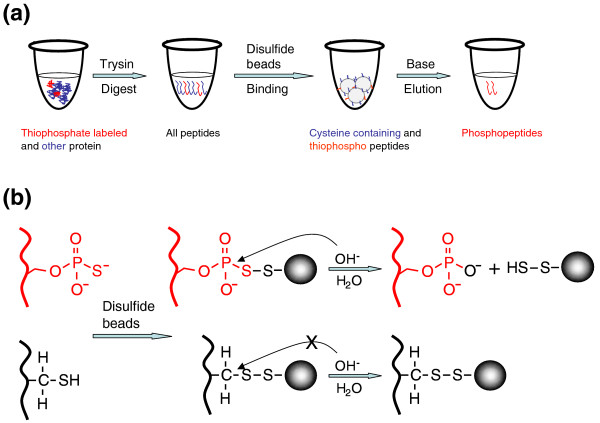
Single-step purification of thiophosphopeptides. **(a) **General scheme for thiophosphospeptide isolation. Proteins were labeled with cyclin A-CDK2 (F80A) and PE-ATP-γ-S and subjected to tryptic digest. The resulting peptides were mixed with disulfide beads, which capture both thiophosphopeptides and cysteine-containing peptides. The beads were then treated with basic solution to selectively release only the phosphopeptides. **(b) **The chemistry underlying the thiophosphopeptide selectivity. Both thiophosphate and cysteine moieties contain reactive thiol groups that can be covalently captured by disulfide beads. At high pH values, the phosphorothiolatesulfide linkages (near the upper arrow) are hydrolyzed to allow the release of the bead-bound peptides while the alkyldisulfide linkages (near the lower arrow) are stable and thus peptides are retained on the beads. Note that during the hydrolysis of the phosphorothiolatesulfide linkage, thiophosphate is converted to normal phosphate.

The use of engineered CDKs and ATP analogues facilitates highly specific substrate phosphorylation: the next challenge is how to identify them within a complex lysate. We sought to utilize the thiophosphate tags to covalently capture and enrich thiophosphorylated peptides after phosphorylation and digestion of the lysates. However, a key problem was to chemically distinguish thiophosphopeptides and cysteine-containing peptides. Although a chemoselective method for enriching thiophosphopeptides has been described, the overwhelming abundance of cysteine residues in a complex protein mixture makes this approach difficult [20]. We used a simple capture-and-release method to selectively isolate thiophosphorylated peptides within trypsinized cell lysates (Figure 2a). Proteins within the lysate were phosphorylated *in vitro *with cyclin A-CDK2 (F80A) and PE-ATP-γ-S. The protein mixture was subsequently digested and the resulting peptides were mixed with Thiopropyl Sepharose 6B [21], an activated disulfide resin that captures both cysteine-containing peptides and thiophosphopeptides through a disulfide exchange reaction. Because traditional dithiothreitol (DTT) elution releases both types of bound peptides, we utilized the qualitative differences between resin-bound thiophosphate peptides and cysteine-containing peptides at the phosphorothiolatesulfide linkage and alkyldisulfide linkage, respectively. At high pH values, the phosphorothiolatesulfide linkage is hydrolyzed and the alkyldisulfide remains intact [22]. Treatment of the resin with a strong base (such as sodium hydroxide) specifically releases thiophosphopeptides (and also converts them to normal phosphopeptides), but not the cysteine-containing peptides, by hydrolyzing the phosphorothiolatesulfide linkage (Figure 2b). Because peptides bound via cysteine are not eluted in the final step, we cannot recover cysteine-containing thiophosphopeptides. Moreover, the elution results in loss of the thiophosphate signature by converting the thiophosphopeptide to a phosphopeptide. Our thiophosphopeptide isolation method differs modestly from that of Blethrow *et al*. [18] in that we capture the thiophosphopeptides using disulfide exchange chemistry (disulfide resin) instead of alkylation (iodoacetamide resin), and we selectively elute them with base hydrolysis rather than oxidation.

To test the feasibility of this approach, we phosphorylated GST-Rb with cyclin A-CDK2 (F80A) and PE-ATP-γ-S, and applied our purification procedure to a trypsin digest of the reaction mixture. The isolated peptides were analyzed by electrospray tandem mass spectrometry (ESI-MS/MS) using an ion trap mass spectrometer. Peptides were identified by matching the tandem mass spectra to a human protein sequence database (with GST-Rb sequence added) using SEQUEST software [23]. The GST-Rb substrate contains five cysteines as well as seven SP/TP sites, which are sites favored for CDK phosphorylation [24]. We recovered multiple phosphopeptides containing only and all the expected phosphorylation sites (Table 1). Furthermore, we recovered no cysteine-containing peptides and very few non-specific peptides. This provided a proof-of-principle for our large-scale assays.

**Table 1:** Phosphopeptides identified from *in vitro *phosphorylated GST-Rb

Peptide sequence	SP/TP sites
R.GSTRPP**T**LS*PIPHIPR.S^†^	S780
R.GSTRPPTLSPIPHIPRS*PYK.F^†^	S788
R.SPYKFPSS*PLR.I	S795
R.IPGGNIYIS*PLKSPYK.I	S807
R.IPGGNIYISPLKS*PYK.I	S811
R.IPGGNIYIS*PLKS*PYK.I	S807, S811
K.ISEGLPT*P**T**K.M	T821
K.ISEGLPT*P**T**KMTPR.S	T821
K.ISEGLPTPTKMT*PR.S	T826
K.ISEGLPT*PTKMT*PR.S	T821, T826

Phosphorylation sites identified with highest probability are marked by boldfaced letters and asterisks to their right. The other boldfaced serine(s) and threonine(s) within the same peptide are potential alternative phosphorylation sites for the ones marked with the asterisk due to the ambiguity in assigning the exact site(s) of phosphorylation. ^†^These are unnatural peptides containing partial sequences of Rb protein and GST fusion.

### Identification of human cyclin A-CDK2 substrates in cell lysates

Our goal was to identify potential cyclin A-CDK2 substrates on a proteome-wide scale. To reduce the sample complexity, we fractionated the whole cell lysate of HEK293 cells into 11 fractions using ion-exchange chromatography and ammonium sulfate precipitation (Additional data file 1). We then carried out *in vitro *kinase assays on each fraction. As a positive control, we also added a small amount of GST-Rb to each reaction. After digesting the reaction mixture with trypsin, we applied our purification protocol to isolate the thiophosphopeptides from the peptide mixtures. The recovered peptides were subjected to liquid chromatography-MS/MS analysis and database searching. We recovered varying numbers of peptides and at least one Rb phosphopeptide from each of the lysate fractions (Additional data file 2).

**Figure 3:**
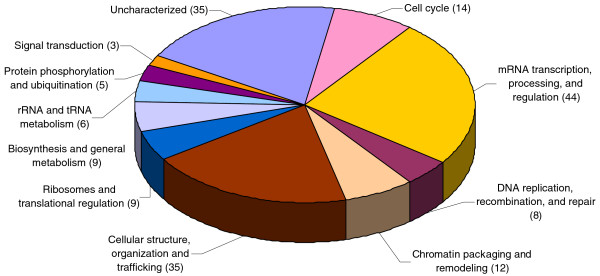
Classification of proteins by functional category. Numbers indicate identified proteins in each category.

CDKs phosphorylate proteins in a proline-directed manner on either serine or threonine, and numerous studies support the idea that the motif S/T-P-X-R/K represents the CDK consensus motif. From the phosphopeptides we identified a total of 203 proteins: 180 candidates were phosphorylated within SP or TP motifs (proline-directed; Additional data file 3). These candidate substrates represent a wide range of biological processes, including cell cycle control, DNA and RNA metabolism, translation and cellular structures (Figure 3). A total of 96 out of 222 (43%) of the proline-directed sites conformed to the known CDK consensus (with a positively charged residue in the +3 position). Interestingly, about 24% of the proline-directed sites (53/222) contained a positively charged residue in either the +4 or +5 positions, suggesting that this motif may also be favored by CDK2. Indeed, Blethrow *et al*. [18] also noted that a substantial number of cyclin B-CDK1 substrates contain non-consensus sites. For the peptides that contained non-consensus sites, we found that about 50% of the corresponding proteins carried at least one K/RXLφ or K/RXLXφ motif (where φ is a large hydrophobic residue and X is any amino acid) distal to the phosphorylation sites, and almost all of them carried at least one minimal RXL motif (Additional data file 3). This is consistent with the well-established idea that these motifs promote cyclin A-CDK2 binding to substrates [16]. In addition to selecting for phosphorylations with CDK consensus motifs, we identified 28 proteins that have been previously implicated as CDK substrates (marked in bold in Additional data file 3) [25-52]. Thus, nearly 15% of our candidates have been previously found as CDK targets, further supporting the idea that our methods captured and enriched for CDK2 substrates. Finally, 43% of the phosphorylations we found have been previously identified in large-scale, *in vivo *phosphoproteome analyses, indicating that these phosphorylations are not limited to our *in lysate *conditions [53-58].

Both our studies and those reported by Blethrow *et al*. [18] used similar phosphopeptide isolation schemes and related cyclin-CDKs, and we anticipated that there might be substantial overlap in the substrates revealed by both studies. Indeed, we found nearly 50% (30/68) of the cyclin B-CDK1 substrates in our list of cyclin A-CDK2 candidates; thus, these methods are robust and reproducible (Additional data file 4). However, there are also substantial differences between the two lists, and these likely resulted from many factors, including procedural differences, different cell types, incomplete peptide identification by MS, and substrate specificity conferred by the cyclin and/or kinase subunits. Some of these differences may also reflect the different biological functions of cyclin A-CDK2 and cyclin B-CDK1. For example, we found nine proteins involved in protein translation and/or ribosome function, but none of these proteins were found with cyclin B-CDK1, despite their relatively high abundance.

Although we identified a number of known CDK2 substrates, we did not identify some previously described CDK2 substrates. Some of the factors listed above may also account for the failure to find known CDK2 substrates in our analyses. In addition, substrates already phosphorylated by endogenous CDKs would not have been thiophosphorylated *in vitro*. It is also possible that some proteins were not solubilized during lysate preparation and/or the sonication step and excluded from our analyses. Finally, it is possible that large protein complexes may have been disrupted by the fractionation procedures prior to the kinase reaction, and that proteins that are phosphorylated by CDK2 only in the context of these complexes may not be discovered by our methods.

We also recovered four phosphopeptides corresponding to cyclin A and CDK2 and 27 additional phosphopeptides with non-proline directed sites (Additional data file 5). We suspected these phosphopeptides resulted from auto-phosphorylation of cyclin A-CDK2 and background phosphorylation by other kinases, and others have reported similar background phosphorylation [15]. To test these possibilities, we carried out a control kinase reaction using cyclin A-CDK2 (F80A) and PE-ATP-γ-S with no cell lysate added and recovered three of the four cyclin A-CDK2 peptides (Additional data file 5). Furthermore, when we performed a similar 'kinase-only' reaction in the presence of γ-^32^P-ATP, we observed ^32^P incorporation into both of these proteins in a dose-dependent manner (Additional data file 6). These experiments confirmed that there was background auto-phosphorylation of cyclin A-CDK2 in the original assays.

We also performed control kinase reactions using the lysate fractions, GST-Rb 'spike-in', and PE-ATP-γ-S without the addition of cyclin A-CDK2. These 'no-kinase' control reactions phosphorylated 7 of the 27 non-proline directed phosphopeptides on our list (Additional data file 5), suggesting that most, if not all, of these phosphopeptides resulted from background phosphorylation by kinases that are able to use the ATP analogue to a limited extent. For example, most of these peptides contain acidic residue-directed phosphorylation sites that are casein kinase 2 motifs. Casein kinase 2 is unique in that it can utilize GTP as well as ATP; thus, the active site may accommodate the bulky ATP analogues, such as PE-ATP [59]. Importantly, we did not recover any Rb phosphopeptides from these control experiments, indicating that there was no non-specific CDK activity in our assays. We also recovered 44 unmodified peptides, 12 of which contained cysteine residues. The majority of these peptides originated from several lysate fractions (Additional data file 2). We suspect these resulted from low-level non-specific binding of peptides to the resin despite stringent wash conditions, and in the case of the cysteine-containing peptides, from a small amount of hydrolysis of the alkyldisulfide linkage during the elution step. In summary, our methods were highly selective, and our studies identified a surprisingly large group of candidate cyclin A-CDK2 substrates, most of which have not been previously identified as CDK targets.

### Validation of candidate substrates as cyclin A-CDK2 targets

**Figure 4:**
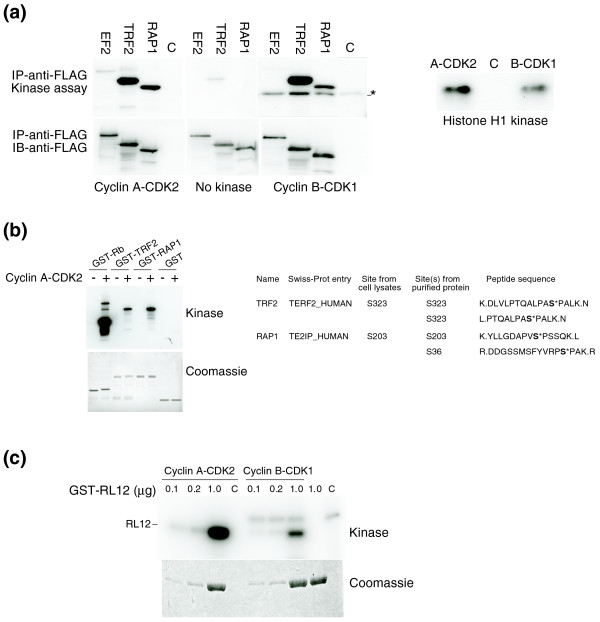
*In vitro *validation of selective candidate CDK2 substrates. **(a) **HEK293 cells were transiently transfected with vectors expressing FLAG-tagged EF2, TRF2, and RAP1. Anti-FLAG antibody immunoprecipitates were *in vitro *phosphorylated with cyclin A-CDK2 or cyclin B-CDK1 in the presence of γ-^32^P-ATP (upper left panels). In parallel reactions, histone H1 was phosphorylated as a control to normalize the activities of cyclin A-CDK2 and cyclin B-CDK1 (right panel). 'C' denotes 'kinase only' reactions without transfected substrates (left panel) and 'no kinase' reaction (right panel). Protein samples were separated by SDS PAGE and the gels were transferred onto PVDF membranes. Phospho-signals were visualized by autoradiography. The membrane was subsequently probed with anti-FLAG antibody (Sigma-Aldrich) to confirm the identity of the phospho-signal bearing band (lower left panels). The asterisk represents a non-specific band from the commercial cyclin B-CDK2 preparation. **(b) **Kinase reaction was carried out using γ-^32^P-ATP, and GST-TRF2, GST-RAP1, GST-Rb (positive control) and GST (negative control) as substrates in the presence or absence of wild-type cyclin A-CDK2 kinase. Reactions were visualized by SDS PAGE followed by Coomassie staining and autoradiography (left panel). A similar kinase assay was carried out using wild-type cyclin A/CDK2 and ATP-γ-S, and subsequently subjected to a phosphopeptide isolation scheme. MS analysis confirmed that TRF2 and RAP1 were each phosphorylated on the exact sites we identified from the screen with one additional site for RAP1 (right panel)**. (c) **Kinase assay was carried out using γ-^32^P-ATP, cyclin A-CDK2 or cyclin B-CDK1 with increasing amounts of purified GST-RL12. Samples were separated by SDS PAGE and the gel was stained with Coomassie (lower panel) followed by autoradiography (upper panel). 'C' denotes 'kinase only' reactions without transfected substrates.

We employed several strategies to validate some of the novel candidates in our list as cyclin A-CDK2 substrates. Because our protein identifications were based on peptide sequences, we began by confirming that cyclin A-CDK2 phosphorylated three full-length and native candidates that were immunoprecipitated via epitope tags from transfected 293 cells (EF2, TRF2, and RAP1). Because these proteins were not present on the cyclin B-CDK1 list [18], we determined if they are also phosphorylated by cyclin B-CDK1. Each CDK2 candidate was phosphorylated by both cyclin A-CDK2 and cyclin B-CDK1, although EF2 was phosphorylated to a lesser extent than either TRF2 or RAP1 (Figure 4a). We also expressed TRF2, RAP1, and the ribosomal protein RL12 as GST-fusions and purified them from *Escherichia coli*. When we used cyclin A-CDK2 to phosphorylate TRF2 and RAP1 *in vitro*, the proteins were highly phosphorylated, and MS analyses revealed that these phosphorylations occurred on the same sites we initially identified (Figure 4b). We also used cyclin A-CDK2 and cyclin B-CDK1 to phosphorylate GST-RL12, and found that both CDKs also phosphorylated RL12 *in vitro *(Figure 4c). These studies thus confirm that the peptides identified in our screen represent proteins that can be phosphorylated by cyclin A-CDK2, at least *in vitro*. Although we did find qualitative differences in the ability of cyclin A-CDK2 and cyclin B-CDK1 to phosphorylate specific proteins, in each case the candidates were phosphorylated by both CDKs. Because the enzyme preparation we used in these studies contained an excess of free CDK2 (F80A), we considered the possibility that some substrate phosphorylations might result from the association of endogenous cyclins with CDK2 (F80A) that was either monomeric, or that may have dissociated from cyclin A during the assay conditions. We found that the amount of cyclin B-CDK2 (F80A) activity in these extracts was negligible compared with cyclin A-CDK2 (F80A), and we could not detect any cyclin E-CDK2 (F80A) activity (Additional data file 7). Nonetheless, we cannot exclude the possibility that some peptides may have been phosphorylated by CDK2 (F80A) in complex with an endogenous cyclin, and the specificity of any candidate substrate for cyclin A versus other cyclins that activate CDK2 needs to be validated as described below.

### Validation of RL12 as an *in vivo *CDK2 substrate

**Figure 5:**
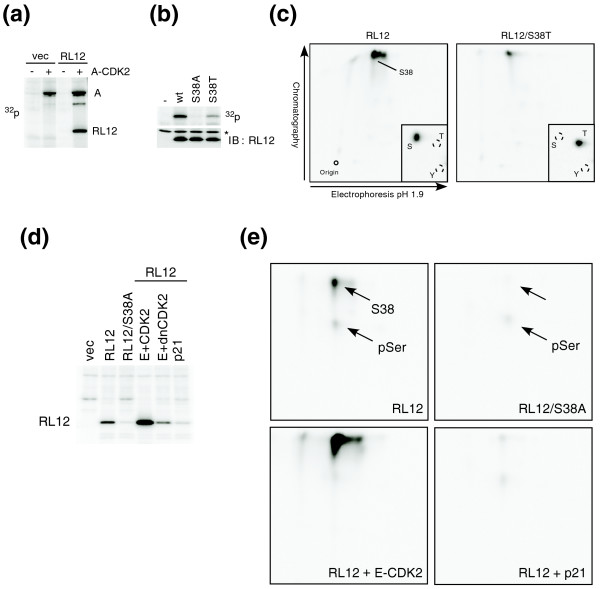
Phosphorylation of RL12 *in vitro *and *in vivo*. **(a) **HA-tagged RL12 or vector control ('vec') were transiently transfected into U2OS cells and immunoprecipitated using 12CA5 antibody. Cyclin A-CDK2 complexes were also transiently expressed separately in U2OS cells and immunoprecipitated using an antibody against cyclin A. Kinase assays were carried out using the RL12 (or control) immunoprecipitate with or without the cyclin A-CDK2 immunoprecipitate in the presence of γ-^32^P-ATP. **(b) **Similar assays were conducted using cyclin A-CDK2 and RL12 immunoprecipitates containing wild-type (wt) and the indicated RL12 phosphosite mutants. The asterisk denotes the light chain of the antibody. **(c) **Phosphopeptide mapping and phosphoamino acid analysis of the radiolabeled wild-type (left panel) and S38T mutant (right panel) of RL12. **(d) **Wild-type RL12, RL12-S38A, or vector control was transiently co-transfected with cyclin E-CDK2, catalytically inactive (dn) cyclin E-CDK2, or p21. All cells were subjected to ^32^P orthophosphate labeling. RL12 was immunoprecipitated from cell lysates and visualized by SDS PAGE followed by autoradiography. **(e) **Phosphopeptide mapping analysis was also carried out on the radiolabeled RL12 shown in (d). The bottom arrow shows a second and minor phosphorylation site detected *in vivo*.

The above studies validated several novel candidates identified in our screen as CDK2 substrates *in vitro*. However, to determine if a novel substrate is also phosphorylated by CDK2 *in vivo*, we performed a more comprehensive analysis of the ribosomal protein RL12. We first mixed immunoprecipitates of epitope-tagged cyclin A-CDK2 and RL12 expressed in human cells in the presence of γ-^32^P-ATP and found that cyclin A-CDK2 phosphorylated RL12 *in vitro *(Figure 5a). The phosphorylation was largely abolished in a mutant RL12 where the identified phosphoserine S38 was replaced with an alanine, and it was restored when S38 was replaced with a threonine (Figure 5b). We then used phosphopeptide mapping to identify the peptide containing S38, and confirmed that it was directly phosphorylated by CDK2 *in vitro *(Figure 5c). To test if RL12 is also phosphorylated *in vivo *in a CDK2-dependent manner at the same site, we metabolically labeled cells with ^32^P-orthophosphate, and immunoprecipitated wild-type or RL12-S38A from cells overexpressing either cyclin E-CDK2, catalytically inactive cyclin E-CDK2, or the CDK inhibitor p21 (to inhibit endogenous CDKs) [60]. Because we cannot study endogenous RL12 phosphorylation due to the lack of a suitable anti-RL12 antibody, these studies examined the phosphorylation of ectopic RL12 *in vivo *(these transfection conditions led to an approximately five- to ten-fold overexpression of RL12 mRNA (Additional data file 8). We found that wild-type RL12, but not RL12-S38A, was phosphorylated *in vivo *(Figure 5d). This phosphorylation was enhanced in cells overexpressing cyclin E-CDK2, but not inactive cyclin E-CDK2, and was diminished in cells overexpressing the p21 CDK inhibitor (which inhibits endogenous cyclin-CDKs; Figure 5d). Finally, we used phosphopeptide mapping and phosphoamino acid analyses of RL12 protein immunoprecipitated from the labeled cells and confirmed that RL12 phosphorylation by cyclin E-CDK2 *in vivo *also occurred on S38 (Figure 5e). RL12 S38 phosphorylation *in vivo *has also been reported in phosphoproteome studies [54,55].

Although we have validated each of the novel candidates that we have tested thus far by showing that the full-length proteins are phosphorylated by CDK2, some candidates on our list will likely prove not to be physiologically relevant cyclin A-CDK2 substrates. For example, the kinase reactions were performed in lysates, and *in vivo *subcellular compartmentalization may restrict the access of CDK2 to some candidates. Moreover, it is possible that other cellular kinases are either redundant with, or more important than, CDK2 with respect to individual substrates *in vivo*. It is thus critical that candidates be rigorously evaluated in as physiological a context as possible. Towards this end, in ongoing studies we are using a gene targeting approach to mutate a subset of these phosphorylation sites in the endogenous genes to study their physiological significance.

## Conclusions

In summary, we describe a rapid and efficient method to identify candidate CDK substrates in cell lysates. We identified 180 candidate cyclin A-CDK2 substrates and found that our method is robust, sensitive, and capable of identifying novel CDK2 targets. Since most protein kinases have conserved ATP binding domains and the kinetics of thiophosphorylation can be optimized [61], these methods should be broadly applicable to the study of many kinases and their substrate networks. Moreover, thiophosphorylation-based phosphopeptide isolation should also facilitate the mapping of phosphorylation sites within individual proteins or protein complexes *in vitro*.

## Materials and methods

### Reagents, cell culture, recombinant protein expression and purification

All standard chemicals were purchased from Sigma-Aldrich (St. Louis, MO, USA). Triphosphate synthesis was carried out according the method of Ludwig [62]. PE-ATP was synthesized similar to as previously described [63] with *N*^6^-(2-phenylethyl)-adenosine as a precursor. PE-ATP-γ-S was custom synthesized by TriLink Biotechnologies (San Diego, CA, USA). All CDK (CDK2 (F80A) and CDK3 (F80A)) and RL12 phosphosite mutants were generated by site-directed mutagenesis with the Quick Change method (Stratagene, La Jolla, CA, USA). All cDNAs used in this study except TRF2 and RAP1 were generated from human mRNA via RT-PCR, and clones were sequenced. RAP1 cDNA was purchased from Open Biosystems (Huntsville, AL, USA). A TRF2 clone was obtained from Addgene (Addgene plasmid 16066, Cambridge, MA, USA). U2OS and HEK293 cell lines were maintained in Dulbecco's modified Eagle's medium with 10% fetal calf serum and expression of wild-type and mutant CDKs and other proteins in U2OS cells were performed by transient transfections or co-transfections via calcium phosphate precipitation using standard procedures. CDKs expressed in human cells were purified by immunoprecipitation using 12CA5 antibody for HA-tagged Cdk subunits or using antibodies against the cyclin subunit. GST-Rb (GST fused to the carboxy-terminal 156 amino acids of Rb), GST-RL12, GST-TRF2, and GST-RAP1 were expressed in bacteria and purified using standard glutathione resin. Purified cyclin A-CDK2 and cyclin B-CDK1 kinases used in Figure 4 were purchased from New England Biolabs (Beverly, MA, USA).

### Preparation of cyclin A-CDK2 complexes expressed in bacteria

Active and soluble wild-type and F80A cyclin A-CDK2 complexes were produced by co-expressing *Saccharomyces cerevisiae *GST-CAK1, full-length untagged cyclin A and His6-tagged CDK2 in bacteria similar to the commercial version (New England Biolabs). Cyclin A and His6-CDK2 were cloned into the pRSFDuet-1 vector (Novagen, San Diego, CA, USA) and GST-CAK1 was cloned into pGEX-2T vector (GE Healthcare, Piscataway, NJ, USA). All three proteins were co-expressed by transforming an *E. coli *BL21 strain with both plasmids. For large scale preparation, cells expressing F80A cyclin A-CDK2 were grown in 500 ml of LB medium to OD_600 _of 0.8 and isopropyl β-D-thiogalactoside (IPTG) was added to 0.5 mM. Cells were then cultured overnight at 37°C before being harvested by centrifugation. The cell pellet was lysed by incubating with 20 ml lysis buffer (50 mM Tris, pH 7.5, 1 mM DTT, 1 mM MgCl_2_, 25 U/ml Benzonase (Novagen), 2 mg/ml lysozyme (Sigma-Aldrich) and protease inhibitors cocktail (Sigma-Aldrich)) at room temperature for 1 h. After NaCl was added to 250 mM and Triton X-100 to 0.025%, the cell slurry was sonicated followed by centrifugation. The supernatant was dialyzed against phosphate-buffered saline (PBS) before incubation with 3 ml of Ni-NTA resin (Qiagen, Valencia, CA, USA), which was washed sequentially with 15 ml PBS and low imidazole buffer (30 mM Tris, pH 7.5, 150 mM NaCl, 50 mM imidazole), and eluted with 6 ml high imidazole buffer (30 mM Tris, pH 7.5, 150 mM NaCl, 300 mM imidazole).

### Immunoblotting, immunoprecipitation, orthophosphate labeling, and phosphoamino acid analysis

These procedures were described previously [64].

### Preparation and fractionation of 293 native cell lysates

HEK293 cells were grown on 15 cm plates to near confluency and harvested in PBS buffer. Cell pellets were resuspended in hypotonic lysis buffer (50 mM Tris, pH 7.5, 1 mM DTT, 1 mM MgCl_2_, 0.1% Triton X-100, 25 U/ml Benzonase (Novagen), and protease inhibitors cocktail (Sigma-Aldrich)) and incubated at 4°C, followed by sonication in 150 mM NaCl. Cell debris was pelleted by centrifugation and the supernatant was diluted such that the salt concentration was below 25 mM. The whole cell lysate was then loaded onto a SP Sepharose (GE Healthcare) column manually by atmospheric pressure and the flowthrough was collected. After washing the column with loading buffer (30 mM Tris, pH 7.5, 25 mM NaCl, 1 mM DTT), bound proteins were eluted sequentially with load buffer containing 100 mM, 200 mM, 300 mM, 400 mM, and 600 mM NaCl. The flowthrough was loaded onto a Q Sepharose (GE Healthcare) column and similar procedures were carried out to collect flowthrough and elute the column. Proteins from Q Sepharose flowthrough were pelleted by ammonium sulfate precipitation at 60% and resuspended in load buffer. All fractions were concentrated with salt concentration adjusted between 100 and 200 mM by serial dilution and concentration. These fractions were used in the first set of experiments and subsequently dialyzed extensively against a Tris buffer (30 mM Tris, pH 7.5, 150 mM NaCl) to be used in a second set of experiments.

### *In vitro *kinase assays and purification of thiophosphorylated peptides

Kinase assays using kinases purified from human cells have been described previously [64]. For kinase assays using cell lysate fractions (or GST-Rb) and PE-ATP-γ-S, recombinant wild-type and F80A cyclin A-CDK2 complexes purified from *E. coli *(as described above) were used. In each of the lysate reactions, about 100-200 μg of lysate fraction (and 100-200 ng of GST-Rb as positive control) was mixed with approximately 1-2 μg of cyclin A-CDK2 (F80A) complex, 250 mM PE-ATP-γ-S in kinase reaction buffer (40 mM Tris, pH 7.5, 10 mM MgCl_2_, 50 mM NaCl). The reaction was incubated at 30°C for 5 h and the protein mixture was denatured by adding acetonitrile to 15% and digested with trypsin (1/20 mass ratio) at 37°C for at least 6 h. Peptides were then incubated with 20 μl of disulfide beads Thiopropyl Sepharose 6B (GE Healthcare) with rotation at room temperature overnight. The beads were loaded onto a Micro Bio-Spin column (Bio-Rad Laboratories, Hercules, CA, USA), and washed sequentially with 3 ml water, 5 ml of 30% acetonitrile in 0.1% formic acid, 5 ml of 2 M NaCl, and 3 ml water. Beads were collected and incubated with 20 μl of 20 mM NaOH at room temperature for 2 h. The eluate was neutralized and acidified with 1% formic acid to pH 3 for direct analysis. Similar phosphopeptide capturing protocol was carried out for the GST-Rb kinase assay using 5 μg of GST-Rb and 0.5 μg of cyclin A-CDK2 (F80A) complex.

### Mass spectrometry analysis and database search

Phosphopeptides samples were analyzed by microcapillary high performance liquid chromatography-electrospray ionization-MS/MS using an ion-trap mass spectrometer (LCQ, ThermoFinnigan, San Jose, CA, USA). Peptides were pressure-loaded onto a 75 μM × 12 cm self-packed C18 column and resolved by a non-linear gradient of 5-28% acetonitrile containing 0.1% formic acid at the flow rate of 200 nl/minute over the course of 2 h. Tandem spectra acquired were searched against the human NCI database (07.20.2006) using SEQUEST. Search parameters included one tryptic end and differential mass modification to serine and threonine due to phosphorylation. For listing purposes, all entries were manually updated using current Swiss-Prot nomenclature. Search results from two independent experiments on each lysate fraction were pooled and filtered using the statistical tool PeptideProphet [65]. Peptides with probabilities higher than 0.9 (error rate <1.8%) were manually validated to further exclude ones with poor MS/MS spectra before inclusion in the final list.

## Abbreviations

CDK, cyclin-dependent kinase; DTT, dithiothreitol; GST, glutathione-S-transferase; MS, mass spectrometry; MS/MS, tandem MS; PBS, phosphate-buffered saline; PE-ATP, *N*^6^-(2-phenylethyl)-ATP; Rb, Retinoblastoma.

## Authors' contributions

YC designed the method, performed the method validation, in-lysate kinase assays, and substrate validation experiments, carried out MS and data analyses. MW performed the kinase mutagenesis and characterization, ATP analogue synthesis and substrate validation experiments. AAH contributed to substrate validation experiments. JJP contributed to the method design. RA provided MS and software resources. YC, RA, and BEC designed the research project. YC and BEC wrote the manuscript.

## Additional data files

The following additional data are available. Additional data file 1 is a figure showing the scheme of HEK293 cell lysate fractionation. Additional data file 2 is a table listing the numbers of peptides identified in the lysate fractions. Additional data file 3 is a table listing the identified proline-directed phosphopeptide sequences, the corresponding protein names, and their functional categorizations. Additional data file 4 is a table comparing the proteins and phosphopeptides identified for cyclin A-CDK2 and cyclin B-CDK1. Additional data file 5 is a table listing the phosphopeptides with non-proline-directed sites and from cyclin A-CDK2 autophosphorylation. Additional data file 6 is a figure showing the autophosphorylation of cyclin A-CDK2 *in vitro*. Additional data file 7 is a figure showing that cyclin B-CDK2 (F80A) and cyclin E-CDK2 (F80A) complexes that may have formed in the lysate represent a negligible fraction of the total amount of CDK2 (F80A) activity. Additional data file 8 is a figure showing the Northern analysis of RL12 expression.

## Supplementary Material

Additional data file 1 Scheme of HEK293 cell lysate fractionation. Click here for file

Additional data file 2 Numbers of peptides identified in the lysate fractions. Click here for file

Additional data file 3 Identified proline-directed phosphopeptide sequences, the corresponding protein names, and their functional categorizations. Click here for file

Additional data file 4 Proteins and phosphopeptides identified for cyclin A-CDK2 and cyclin B-CDK1. Click here for file

Additional data file 5 Phosphopeptides with non-proline-directed sites and from cyclin A-CDK2 autophosphorylation. Click here for file

Additional data file 6 Autophosphorylation of cyclin A-CDK2 *in vitro*. Click here for file

Additional data file 7 Cyclin B-CDK2 (F80A) and cyclin E-CDK2 (F80A) complexes that may have formed in the lysate represent a negligible fraction of the total amount of CDK2 (F80A) activity. Click here for file

Additional data file 8 Northern analysis of RL12 expression. Click here for file

## References

[B1] Manning G, Whyte DB, Martinez R, Hunter T, Sudarsanam S (2002). The protein kinase complement of the human genome.. Science.

[B2] Cohen P (2000). The regulation of protein function by multisite phosphorylation - a 25 year update.. Trends Biochem Sci.

[B3] Kane S, Sano H, Liu SC, Asara JM, Lane WS, Garner CC, Lienhard GE (2002). A method to identify serine kinase substrates. Akt phosphorylates a novel adipocyte protein with a Rab GTPase-activating protein (GAP) domain.. J Biol Chem.

[B4] Matsuoka S, Ballif BA, Smogorzewska A, McDonald ER, Hurov KE, Luo J, Bakalarski CE, Zhao Z, Solimini N, Lerenthal Y, Shiloh Y, Gygi SP, Elledge SJ (2007). ATM and ATR substrate analysis reveals extensive protein networks responsive to DNA damage.. Science.

[B5] Smolka MB, Albuquerque CP, Chen SH, Zhou H (2007). Proteome-wide identification of *in vivo *targets of DNA damage checkpoint kinases.. Proc Natl Acad Sci USA.

[B6] Ptacek J, Devgan G, Michaud G, Zhu H, Zhu X, Fasolo J, Guo H, Jona G, Breitkreutz A, Sopko R, McCartney RR, Schmidt MC, Rachidi N, Lee SJ, Mah AS, Meng L, Stark MJ, Stern DF, De Virgilio C, Tyers M, Andrews B, Gerstein M, Schweitzer B, Predki PF, Snyder M (2005). Global analysis of protein phosphorylation in yeast.. Nature.

[B7] Shah K, Shokat KM (2003). A chemical genetic approach for the identification of direct substrates of protein kinases.. Methods Mol Biol.

[B8] Liu Y, Shah K, Yang F, Witucki L, Shokat KM (1998). A molecular gate which controls unnatural ATP analogue recognition by the tyrosine kinase v-Src.. Bioorg Med Chem.

[B9] Shah K, Liu Y, Deirmengian C, Shokat KM (1997). Engineering unnatural nucleotide specificity for Rous sarcoma virus tyrosine kinase to uniquely label its direct substrates.. Proc Natl Acad Sci USA.

[B10] Liu Y, Shah K, Yang F, Witucki L, Shokat KM (1998). Engineering Src family protein kinases with unnatural nucleotide specificity.. Chem Biol.

[B11] Habelhah H, Shah K, Huang L, Burlingame AL, Shokat KM, Ronai Z (2001). Identification of new JNK substrate using ATP pocket mutant JNK and a corresponding ATP analogue.. J Biol Chem.

[B12] Eblen ST, Kumar NV, Shah K, Henderson MJ, Watts CK, Shokat KM, Weber MJ (2003). Identification of novel ERK2 substrates through use of an engineered kinase and ATP analogs.. J Biol Chem.

[B13] Shah K, Shokat KM (2002). A chemical genetic screen for direct v-Src substrates reveals ordered assembly of a retrograde signaling pathway.. Chem Biol.

[B14] Dephoure N, Howson RW, Blethrow JD, Shokat KM, O'Shea EK (2005). Combining chemical genetics and proteomics to identify protein kinase substrates.. Proc Natl Acad Sci USA.

[B15] Ubersax JA, Woodbury EL, Quang PN, Paraz M, Blethrow JD, Shah K, Shokat KM, Morgan DO (2003). Targets of the cyclin-dependent kinase Cdk1.. Nature.

[B16] Harper JW, Adams PD (2001). Cyclin-dependent kinases.. Chem Rev.

[B17] Morgan DO (1995). Principles of CDK regulation.. Nature.

[B18] Blethrow JD, Glavy JS, Morgan DO, Shokat KM (2008). Covalent capture of kinase-specific phosphopeptides reveals Cdk1-cyclin B substrates.. Proc Natl Acad Sci USA.

[B19] Hiriyanna KT, Baedke D, Baek KH, Forney BA, Kordiyak G, Ingebritsen TS (1994). Thiophosphorylated substrate analogs are potent active site-directed inhibitors of protein-tyrosine phosphatases.. Anal Biochem.

[B20] Kwon SW, Kim SC, Jaunbergs J, Falck JR, Zhao Y (2003). Selective enrichment of thiophosphorylated polypeptides as a tool for the analysis of protein phosphorylation.. Mol Cell Proteomics.

[B21] Liu T, Qian WJ, Strittmatter EF, Camp DG, Anderson GA, Thrall BD, Smith RD (2004). High-throughput comparative proteome analysis using a quantitative cysteinyl-peptide enrichment technology.. Anal Chem.

[B22] Sengle G, Jenne A, Arora PS, Seelig B, Nowick JS, Jaschke A, Famulok M (2000). Synthesis, incorporation efficiency, and stability of disulfide bridged functional groups at RNA 5'-ends.. Bioorg Med Chem.

[B23] Eng JK, McCormack AL, Yates JR (1994). An approach to correlate tandem mass spectral data of peptides with amino acid sequences in a protein database. J Am Soc Mass Spectrom.

[B24] Songyang Z, Blechner S, Hoagland N, Hoekstra MF, Piwnica-Worms H, Cantley LC (1994). Use of an oriented peptide library to determine the optimal substrates of protein kinases.. Curr Biol.

[B25] Graub R, Lancero H, Pedersen A, Chu M, Padmanabhan K, Xu XQ, Spitz P, Chalkley R, Burlingame AL, Stokoe D, Bernstein HS (2008). Cell cycle-dependent phosphorylation of human CDC5 regulates RNA processing.. Cell Cycle.

[B26] Ferrari G, Rossi R, Arosio D, Vindigni A, Biamonti G, Montecucco A (2003). Cell cycle-dependent phosphorylation of human DNA ligase I at the cyclin-dependent kinase sites.. J Biol Chem.

[B27] Taguchi N, Ishihara N, Jofuku A, Oka T, Mihara K (2007). Mitotic phosphorylation of dynamin-related GTPase Drp1 participates in mitochondrial fission.. J Biol Chem.

[B28] Yamashita N, Morita A, Uchida Y, Nakamura F, Usui H, Ohshima T, Taniguchi M, Honnorat J, Thomasset N, Takei K, Takahashi T, Kolattukudy P, Goshima Y (2007). Regulation of spine development by semaphorin3A through cyclin-dependent kinase 5 phosphorylation of collapsin response mediator protein 1.. J Neurosci.

[B29] Kim HH, Abdelmohsen K, Lal A, Pullmann R, Yang X, Galban S, Srikantan S, Martindale JL, Blethrow J, Shokat KM, Gorospe M (2008). Nuclear HuR accumulation through phosphorylation by Cdk1.. Genes Dev.

[B30] Hale TK, Contreras A, Morrison AJ, Herrera RE (2006). Phosphorylation of the linker histone H1 by CDK regulates its binding to HP1alpha.. Mol Cell.

[B31] Hall C, Nelson DM, Ye X, Baker K, DeCaprio JA, Seeholzer S, Lipinski M, Adams PD (2001). HIRA, the human homologue of yeast Hir1p and Hir2p, is a novel cyclin-cdk2 substrate whose expression blocks S-phase progression.. Mol Cell Biol.

[B32] Ohsugi M, Tokai-Nishizumi N, Shiroguchi K, Toyoshima YY, Inoue J, Yamamoto T (2003). Cdc2-mediated phosphorylation of Kid controls its distribution to spindle and chromosomes.. EMBO J.

[B33] Muller-Tidow C, Ji P, Diederichs S, Potratz J, Baumer N, Kohler G, Cauvet T, Choudary C, Meer T van der, Chan WY, Nieduszynski C, Colledge WH, Carrington M, Koeffler HP, Restle A, Wiesmüller L, Sobczak-Thépot J, Berdel WE, Serve H (2004). The cyclin A1-CDK2 complex regulates DNA double-strand break repair.. Mol Cell Biol.

[B34] Luscher B, Brizuela L, Beach D, Eisenman RN (1991). A role for the p34cdc2 kinase and phosphatases in the regulation of phosphorylation and disassembly of lamin B2 during the cell cycle.. EMBO J.

[B35] Manenti S, Yamauchi E, Sorokine O, Knibiehler M, Van Dorsselaer A, Taniguchi H, Ducommun B, Darbon JM (1999). Phosphorylation of the myristoylated protein kinase C substrate MARCKS by the cyclin E-cyclin-dependent kinase 2 complex *in vitro*.. Biochem J.

[B36] Montagnoli A, Valsasina B, Brotherton D, Troiani S, Rainoldi S, Tenca P, Molinari A, Santocanale C (2006). Identification of Mcm2 phosphorylation sites by S-phase-regulating kinases.. J Biol Chem.

[B37] Komamura-Kohno Y, Karasawa-Shimizu K, Saitoh T, Sato M, Hanaoka F, Tanaka S, Ishimi Y (2006). Site-specific phosphorylation of MCM4 during the cell cycle in mammalian cells.. FEBS J.

[B38] Hirohashi Y, Wang Q, Liu Q, Li B, Du X, Zhang H, Furuuchi K, Masuda K, Sato N, Greene MI (2006). Centrosomal proteins Nde1 and Su48 form a complex regulated by phosphorylation.. Oncogene.

[B39] Bassermann F, von Klitzing C, Illert AL, Munch S, Morris SW, Pagano M, Peschel C, Duyster J (2007). Multisite phosphorylation of nuclear interaction partner of ALK (NIPA) at G2/M involves cyclin B1/Cdk1.. J Biol Chem.

[B40] Okuda M, Horn HF, Tarapore P, Tokuyama Y, Smulian AG, Chan PK, Knudsen ES, Hofmann IA, Snyder JD, Bove KE, Fukasawa K (2000). Nucleophosmin/B23 is a target of CDK2/cyclin E in centrosome duplication.. Cell.

[B41] Ostvold AC, Norum JH, Mathiesen S, Wanvik B, Sefland I, Grundt K (2001). Molecular cloning of a mammalian nuclear phosphoprotein NUCKS, which serves as a substrate for Cdk1 *in vivo*.. Eur J Biochem.

[B42] Peter M, Nakagawa J, Doree M, Labbe JC, Nigg EA (1990). Identification of major nucleolar proteins as candidate mitotic substrates of cdc2 kinase.. Cell.

[B43] Wang Y, Prives C (1995). Increased and altered DNA binding of human p53 by S and G2/M but not G1 cyclin-dependent kinases.. Nature.

[B44] Roberts SB, Segil N, Heintz N (1991). Differential phosphorylation of the transcription factor Oct1 during the cell cycle.. Science.

[B45] Liu CW, Wang RH, Dohadwala M, Schonthal AH, Villa-Moruzzi E, Berndt N (1999). Inhibitory phosphorylation of PP1alpha catalytic subunit during the G(1)/S transition.. J Biol Chem.

[B46] Gebara MM, Sayre MH, Corden JL (1997). Phosphorylation of the carboxy-terminal repeat domain in RNA polymerase II by cyclin-dependent kinases is sufficient to inhibit transcription.. J Cell Biochem.

[B47] Seghezzi W, Chua K, Shanahan F, Gozani O, Reed R, Lees E (1998). Cyclin E associates with components of the pre-mRNA splicing machinery in mammalian cells.. Mol Cell Biol.

[B48] Pei Y, Du H, Singer J, Stamour C, Granitto S, Shuman S, Fisher RP (2006). Cyclin-dependent kinase 9 (Cdk9) of fission yeast is activated by the CDK-activating kinase Csk1, overlaps functionally with the TFIIH-associated kinase Mcs6, and associates with the mRNA cap methyltransferase Pcm1 *in vivo*.. Mol Cell Biol.

[B49] Brattsand G, Marklund U, Nylander K, Roos G, Gullberg M (1994). Cell-cycle-regulated phosphorylation of oncoprotein 18 on Ser16, Ser25 and Ser38.. Eur J Biochem.

[B50] Linding R, Jensen LJ, Ostheimer GJ, van Vugt MA, Jorgensen C, Miron IM, Diella F, Colwill K, Taylor L, Elder K, Metalnikov P, Nguyen V, Pasculescu A, Jin J, Park JG, Samson LD, Woodgett JR, Russell RB, Bork P, Yaffe MB, Pawson T (2007). Systematic discovery of in vivo phosphorylation networks.. Cell.

[B51] Yamaguchi T, Goto H, Yokoyama T, Sillje H, Hanisch A, Uldschmid A, Takai Y, Oguri T, Nigg EA, Inagaki M (2005). Phosphorylation by Cdk1 induces Plk1-mediated vimentin phosphorylation during mitosis.. J Cell Biol.

[B52] Hirota T, Morisaki T, Nishiyama Y, Marumoto T, Tada K, Hara T, Masuko N, Inagaki M, Hatakeyama K, Saya H (2000). Zyxin, a regulator of actin filament assembly, targets the mitotic apparatus by interacting with h-warts/LATS1 tumor suppressor.. J Cell Biol.

[B53] Beausoleil SA, Jedrychowski M, Schwartz D, Elias JE, Villen J, Li J, Cohn MA, Cantley LC, Gygi SP (2004). Large-scale characterization of HeLa cell nuclear phosphoproteins.. Proc Natl Acad Sci USA.

[B54] Beausoleil SA, Villen J, Gerber SA, Rush J, Gygi SP (2006). A probability-based approach for high-throughput protein phosphorylation analysis and site localization.. Nat Biotechnol.

[B55] Olsen JV, Blagoev B, Gnad F, Macek B, Kumar C, Mortensen P, Mann M (2006). Global, *in vivo*, and site-specific phosphorylation dynamics in signaling networks.. Cell.

[B56] Yu LR, Zhu Z, Chan KC, Issaq HJ, Dimitrov DS, Veenstra TD (2007). Improved titanium dioxide enrichment of phosphopeptides from HeLa cells and high confident phosphopeptide identification by cross-validation of MS/MS and MS/MS/MS spectra.. J Proteome Res.

[B57] Molina H, Horn DM, Tang N, Mathivanan S, Pandey A (2007). Global proteomic profiling of phosphopeptides using electron transfer dissociation tandem mass spectrometry.. Proc Natl Acad Sci USA.

[B58] Nousiainen M, Sillje HH, Sauer G, Nigg EA, Korner R (2006). Phosphoproteome analysis of the human mitotic spindle.. Proc Natl Acad Sci USA.

[B59] Niefind K, Putter M, Guerra B, Issinger OG, Schomburg D (1999). GTP plus water mimic ATP in the active site of protein kinase CK2.. Nat Struct Biol.

[B60] Sherr CJ, Roberts JM (1999). CDK inhibitors: positive and negative regulators of G1-phase progression.. Genes Dev.

[B61] Parker LL, Schilling AB, Kron SJ, Kent SB (2005). Optimizing thiophosphorylation in the presence of competing phosphorylation with MALDI-TOF-MS detection.. J Proteome Res.

[B62] Ludwig J (1981). A new route to nucleoside 5'-triphosphates.. Acta Biochim Biophys Acad Sci Hung.

[B63] Kraybill BC, Elkin LL, Blethrow JD, Morgan DO, Shokat KM (2002). Inhibitor scaffolds as new allele specific kinase substrates.. J Am Chem Soc.

[B64] Welcker M, Singer J, Loeb KR, Grim J, Bloecher A, Gurien-West M, Clurman BE, Roberts JM (2003). Multisite phosphorylation by Cdk2 and GSK3 controls cyclin E degradation.. Mol Cell.

[B65] Nesvizhskii AI, Keller A, Kolker E, Aebersold R (2003). A statistical model for identifying proteins by tandem mass spectrometry.. Anal Chem.

