# Supplementary material for: Identification of CDK2 substrates in human cell lysates
Source: Genome Biol. 2008 Oct 13;9(10):R149. doi: 10.1186/gb-2008-9-10-r149 (PMC2760876; doi:10.1186/gb-2008-9-10-r149)
Supplement: Additional data file 1 — Scheme of HEK293 cell lysate fractionation. [file gb-2008-9-10-r149-S1.pdf]

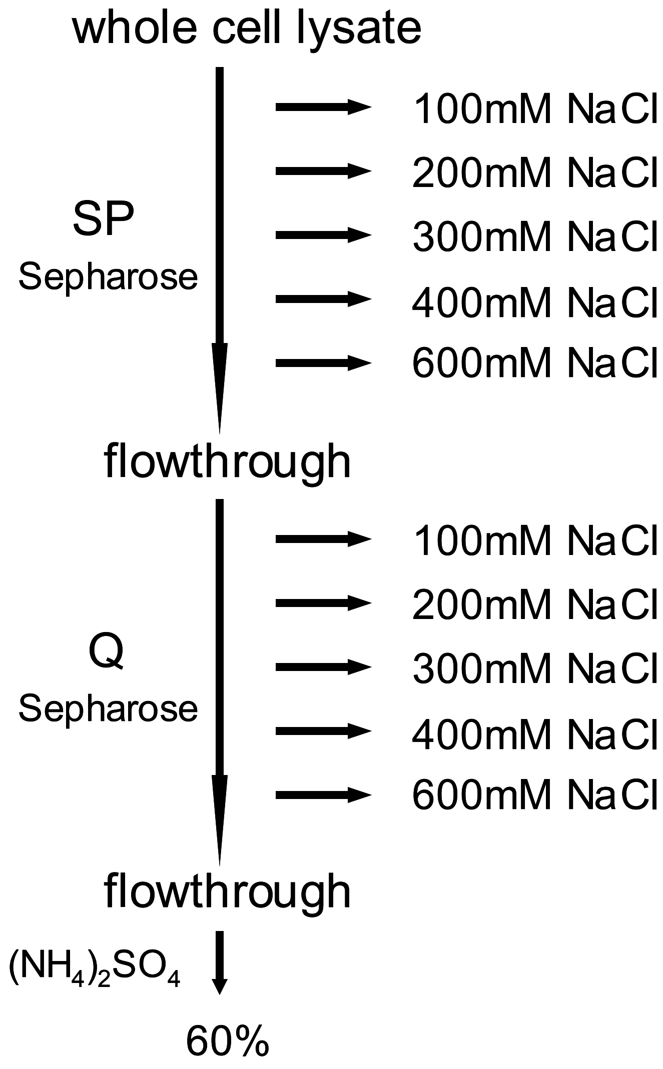

Additional data file 1 - Flow diagram of HEK293 whole cell lysate fractionation
HEK293 whole cell lysate was sequentially subjected to a cation-exchange (SP Sepharose) and anion-exchange (Q Sepharose). Proteins on each column were eluted with salt concentrations indicated, and proteins in the final flowthrough were concentrated by ammonium sulphate precipitation, resulting in 11 fractions.
