# Supplementary material for: Identification of CDK2 substrates in human cell lysates
Source: Genome Biol. 2008 Oct 13;9(10):R149. doi: 10.1186/gb-2008-9-10-r149 (PMC2760876; doi:10.1186/gb-2008-9-10-r149)
Supplement: Additional data file 6 — Autophosphorylation of cyclin A-CDK2 in vitro. [file gb-2008-9-10-r149-S6.pdf]

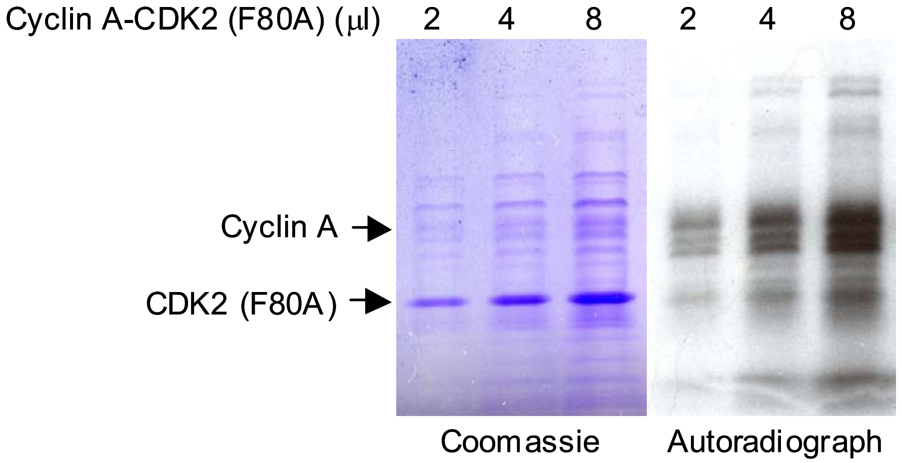

Additional data file 6 - Autophosphorylation of cyclin A-CDK2

Kinase assays were carried out using increasing amounts of cyclin A-CDK2 (F80A) complex in the presence of γ-^{32}P-ATP. The reaction mixtures were fractionated by SDS PAGE, and the gel was Coomassie stained (left panel) and autoradiographed (right panel). Cyclin A and CDK2 bands (arrows) were confirmed by MS sequencing. Note that CDK2 is in large excess compared cyclin A in the kinase preparation.
