# Supplementary material for: Identification of CDK2 substrates in human cell lysates
Source: Genome Biol. 2008 Oct 13;9(10):R149. doi: 10.1186/gb-2008-9-10-r149 (PMC2760876; doi:10.1186/gb-2008-9-10-r149)
Supplement: Additional data file 7 — Cyclin B-CDK2 (F80A) and cyclin E-CDK2 (F80A) complexes that may have formed in the lysate represent a negligible fraction of the total amount of CDK2 (F80A) activity. [file gb-2008-9-10-r149-S7.pdf]

A

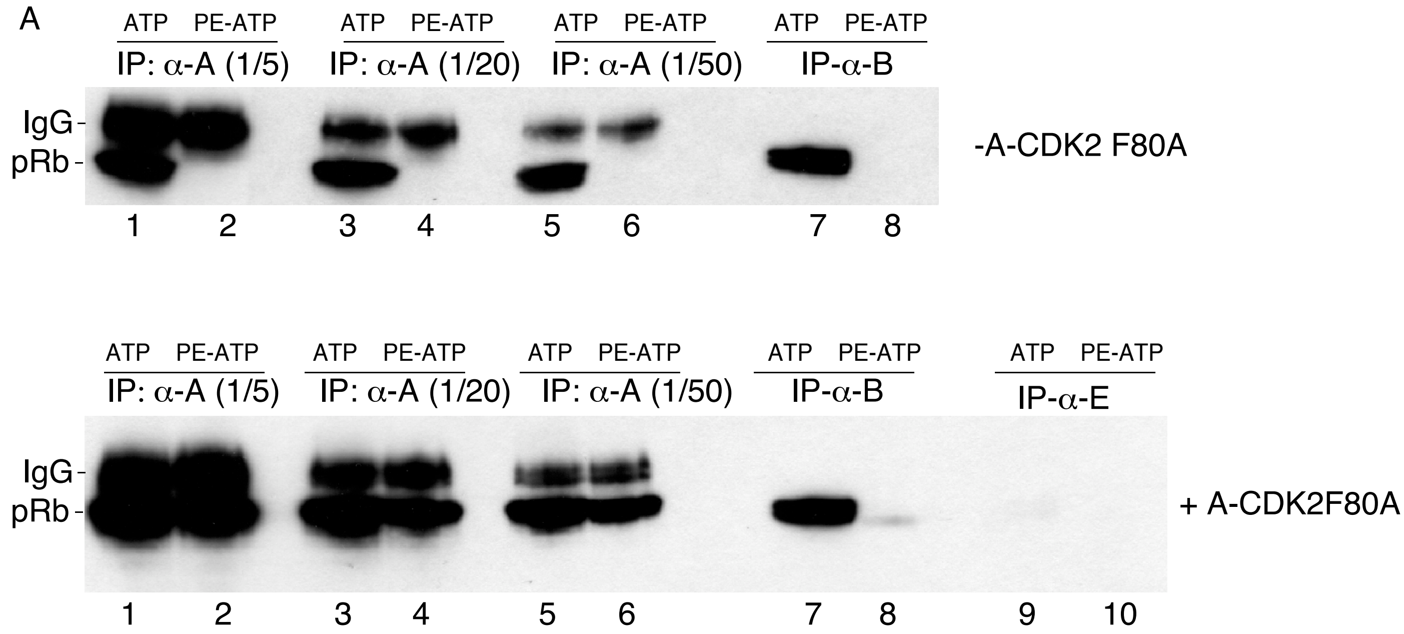

B

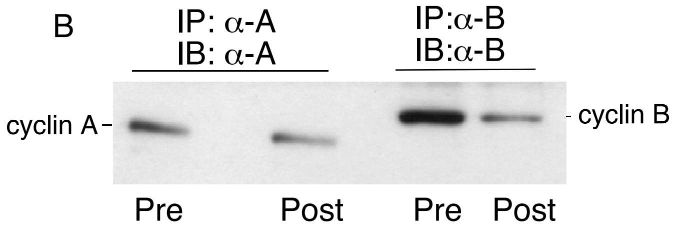

Additional data file 7 - Binding of endogenous cyclins to CDK2 (F80A)

293 whole cell lysates were prepared and cyclin A-CDK2 (F80A) added as described in the Materials and methods for the kinase reactions. A. Cyclin A was immunodepleted with the indicated amount of polyclonal anti-cyclin A antibody (e.g. 1/5 = 0.2 μl). Cyclin B and cyclin E were immunoprecipitated with monoclonal antibodies as indicated. The immunoprecipitates were washed and used to phosphorylate a GST-Rb substrate, and the amount of Rb phosphorylation was determined by western blotting with a rabbit polyclonal anti-phospho-Rb antibody. Reactions were preformed in the presence of ATP or PE-ATP as indicated. The top panel shows that in the absence of added cyclin A-CDK2 (F80A), there is no detectable Rb kinase activity when PE-ATP is used. The bottom panel shows the amount of Rb kinase activity present in the immunoprecipitates from lysates after addition of cyclin A-CDK2 (F80A). Note the high levels of Rb-kinase activity in the cyclin A immunoprecipitates (lanes 2, 4, 6) compared with either cyclin B (lane 8) or cyclin E (lane 10). The decreasing amounts of anti-cyclin A antibody were used because of heavy signal interference from the IgG band (see below). B. Immunodepletion of cyclins in panel A. Samples were taken from the lysate before and after the immunodepletion. In this case 1.0 μl of anti-cyclin A antibody was used. At this exposure all of the cyclin A signal detected is due to the ectopic cyclin A (not shown) and no appreciable cyclin A immunodepletion was seen. Thus the amount of cyclin A-associated kinase activity shown in panel A represents only a very small fraction of the amount of cyclin A-CDK2 (F80A) activity present in the lysate, since smaller amounts of anti-cyclin A antibody were used. In contrast, the cyclin B immunoprecipitation depleted most of the cyclin B from the lysate. These data indicate that cyclin B-CDK2 (F80A) complexes that may have formed in the lysate represent an extremely small fraction of the total amount of CDK2 (F80A) activity.
