# Supplementary material for: Identification of CDK2 substrates in human cell lysates
Source: Genome Biol. 2008 Oct 13;9(10):R149. doi: 10.1186/gb-2008-9-10-r149 (PMC2760876; doi:10.1186/gb-2008-9-10-r149)
Supplement: Additional data file 8 — Northern analysis of RL12 expression. [file gb-2008-9-10-r149-S8.pdf]

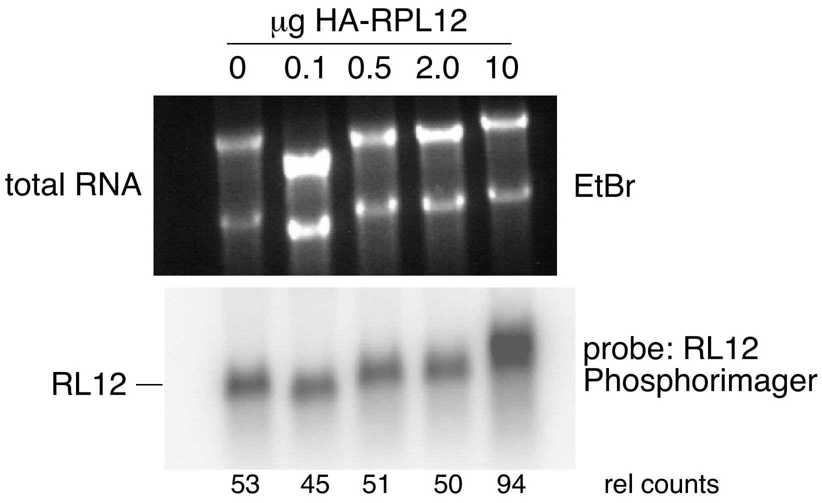

Additional data file 8 - Northern analysis of RL12 expression

U2OS cells were transfected with the indicated amount of RL12 expression plasmid and empty vector to keep the DNA amount constant at 10 μg. Total RNA was isolated 48 hours after transfection, and 10 μg of RNA was run on a formaldehyde-agarose gel and transferred to PVDF. The top panel shows the total RNA present in the gel prior to transfer. The bottom panel shows the filter probed with a radiolabeled RL12 probe and exposed on a phosphorimager for 20 h. The quantitation of the band is shown below the filter image. The 10 μg transfection resulted in approximately a two-fold elevation in RL12 mRNA and the transfection efficiency was 25%, as determined by co-transfection with a GFP expression plasmid (0.5 μg/plate).
